# How to set the bar in competency-based medical education: standard setting after an Objective Structured Clinical Examination (OSCE)

**DOI:** 10.1186/s12909-015-0506-z

**Published:** 2016-01-04

**Authors:** Tim Dwyer, Sarah Wright, Kulamakan Mahan Kulasegaram, John Theodoropoulos, Jaskarndip Chahal, David Wasserstein, Charlotte Ringsted, Brian Hodges, Darrell Ogilvie-Harris

**Affiliations:** Women’s College Hospital, 76 Grenville St, Toronto, M5S 1B1 Canada; Mt Sinai Hospital, 600 University Avenue, Toronto, M5G 1X5 Canada; Toronto East General, 825 Coxell Avenue, Toronto, M4C 3E7 Canada; The Wilson Centre, 200 Elizabeth St, Toronto, M5G 2C4 Canada; Sunnybrook Health Sciences Centre, 2075 Bayview Ave, Toronto, M4N 3 M5 Canada

## Abstract

**Background:**

The goal of the Objective Structured Clinical Examination (OSCE) in Competency-based Medical Education (CBME) is to establish a minimal level of competence. The purpose of this study was to 1) to determine the credibility and acceptability of the modified Angoff method of standard setting in the setting of CBME, using the Borderline Group (BG) method and the Borderline Regression (BLR) method as a reference standard; 2) to determine if it is feasible to set different standards for junior and senior residents, and 3) to determine the desired characteristics of the judges applying the modified Angoff method.

**Methods:**

The results of a previous OSCE study (21 junior residents, 18 senior residents, and six fellows) were used. Three groups of judges performed the modified Angoff method for both junior and senior residents: 1) sports medicine surgeons, 2) non-sports medicine orthopedic surgeons, and 3) sports fellows. Judges defined a borderline resident as a resident performing at a level between competent and a novice at each station. For each checklist item, the judges answered yes or no for “will the borderline/advanced beginner examinee respond correctly to this item?” The pass mark was calculated by averaging the scores. This pass mark was compared to that created using both the BG and the BLR methods.

**Results:**

A paired *t*-test showed that all examiner groups expected senior residents to get significantly higher percentage of checklist items correct compared to junior residents (all stations *p* < 0.001). There were no significant differences due to judge type. For senior residents, there were no significant differences between the cut scores determined by the modified Angoff method and the BG/BLR method. For junior residents, the cut scores determined by the modified Angoff method were lower than the cut scores determined by the BG/BLR Method (all *p* < 0.01).

**Conclusion:**

The results of this study show that the modified Angoff method is an acceptable method of setting different pass marks for senior and junior residents. The use of this method enables both senior and junior residents to sit the same OSCE, preferable in the regular assessment environment of CBME.

## Background

One of the key components of competency-based medical education (CBME) is regular assessment. At our institution, which has been trialing CBME in the postgraduate setting since 2009, the division of orthopedics has been using an end of rotation Objective Structured Clinical Examination (OSCE) to determine if residents are competent to progress to the next rotation - the goal of this in-training OSCE is to establish a minimal level of competence [1].

Under the CBME curriculum at our university, residents undertake the sports medicine rotation twice, once as a junior resident (postgraduate year (PGY) 1–3), and once as a senior resident (PGY 4&5). While the curriculum map details different goals for junior and senior residents, for issues of feasibility all residents have been sitting the same OSCE. It was also believed that exposure of junior residents to the expected standard of clinical performance was important.

A previous study examining the use of an OSCE after a three months sports medicine rotation in a CBME program studied 45 participants (21 junior, 18 senior, six fellows), who undertook a six station OSCE over an 18-month period (unpublished data). The results of this OSCE (reliability >0.8), which tested the application of clinical skills, demonstrated a significant difference between junior and senior residents for the overall global ratings, total checklist scores, as well as for the global ratings/checklist scores for each station. Using a non-compensatory method (residents had to be deemed competent in 4/6 stations), only 8/21 (38 %) of junior residents passed the exam, while 18/18 (100 %) of senior residents passed.

Clearly, a pass rate of 38 % for the junior residents appears unacceptable. Inherent in the two rotations for sports medicine is a belief that junior residents cannot become competent at sports medicine in a single rotation. Furthermore, different objectives had been set for the junior residents in the curriculum. In order to continue using a single OSCE, it was felt that a criterion-referenced standard setting method could be applied, whereby cut-scores would be set on the basis of what the resident should know, most suitable in the setting of CBME [2–5].

Medical schools commonly use an examinee-centered standard setting method such as the mean Borderline Group (BG) method [6–8]. This method involves examiners in each station giving a global rating of each student’s overall performance independent of the checklist mark - the mean of scores for all candidates identified as borderline becomes the pass mark for the station, with the pass mark for the whole OSCE calculated by averaging each station’s borderline score [9, 10]. An alternative method used in the setting of small numbers of candidates (such as postgraduate orthopedic training) is the Borderline Regression (BLR) method, which predicts total OSCE scores from global ratings using linear regression, calculating the pass mark by substituting the score of borderline candidates into the regression equation for each OSCE [2, 11, 12]. The downside of such methods in CBME is that the pass mark must be set *after* the exam has been undertaken.

An alternative method is a test-centered method whereby the pass mark is based on item or station characteristics, such as the modified Angoff standard setting method. In the modified Angoff methods, judges reviewed each question after defining a borderline candidate, and decided whether the borderline examinee will respond correctly [2, 6, 13, 14]. The main advantage of such a method is that the pass/fail standard can be reliably set *before* the OSCE is undertaken, which would be useful in the setting of CBME [2, 15, 16]. Furthermore, a modified Angoff could potentially be used to set different pass marks for junior and senior residents, eliminating the need for two end of rotation OSCEs.

The purpose of this study was to 1) to determine the credibility and acceptability of the modified Angoff method of standard setting in the setting of CBME, using the BG and the BLR method as a reference standard; 2) to determine if it is feasible to set different standards for junior and senior residents, and 3) to determine the desired characteristics of the judges applying the modified Angoff method.

## Methods

### The modified Angoff method

In order to determine the most appropriate judges to establish competence in the setting of an orthopedic sports medicine rotation, three groups of judges were used: 1) sports medicine subspecialty surgeons, 2) non-sports medicine orthopedic surgeons, and 3) newly graduated orthopedic surgeons/orthopedic fellows. Six judges from each group participated, with this number based upon previous research [17–19]. All judges, with the exception of the fellows, were members of faculty, experienced at both teaching and examining orthopedic residents – no judge had previous experience with standard setting methods.

Prior to the start of the OSCE, judges were asked to define an advanced beginner/borderline resident as a resident who is performing at a level between novice and competent at each station. For each checklist item for each station, the judges answered yes or no for “will the borderline examinee respond correctly to this item?” [2, 14]. Items were then assigned as yes = 1 and no = 0, and the pass mark calculated by averaging the scores [2]. Judges were asked to perform the modified Angoff method while separately imaging a borderline senior resident in PGY 4&5, and a borderline junior resident in PGY 1–3. The lead author discussed the modified Angoff method individually with each judge, and participated in the evaluation of the first 10 checklist questions on random stations.

### The borderline group method

After the conclusion of the study using an OSCE to examine the performance of residents after the sports rotation, the residents who were rated advanced beginner for each station were called the borderline group for that station. The mean checklist score of this group was calculated and used as the station pass mark [6–10].

### The Borderline Regression (BLR) method

The Borderline Regression (BLR) method was used to calculate pass marks based on the results of a regression analysis, using the global station mark as the independent variable and the station checklist mark as the dependent variable [2, 12, 20]. The regression line was calculated based on all of the interactions between examiners and students for a particular station. The pass mark was then the point at which the regression line crosses the borderline category. The overall pass mark using BLR method was calculated as the total of all of the station pass marks plus 1 Standard Error of Measurement (SEM) (roughly equivalent to 0.5*SD of the overall score). The SEM is calculated by taking the square root of (1-cronbach’s alpha for checklist total)* SD.

### Ethical consideration

Approval for this study was obtained from Women’s College Hospital Research Ethics Board, approval number 2012-0009-E. All participants consented to the use of their de-identified data.

### Statistical analysis

The mean passing mark, pass rates (%), confidence intervals, and SEMs were calculated for each of the methods, for each station and overall. Differences between pass/fail standards of the modified Angoff, the BG, and the BLR methods were tested using a paired *t*-test, considering *p* < 0.05 as statistically significant [20]. The inter-rater judge agreement for the Angoff method was calculated for each item, station and overall, using an intra-class correlation coefficient. The acceptability (satisfactoriness) or impact of each standard setting method on exam level pass/fail decisions was examined, based upon the passing rates (percentage of residents passing) of the junior residents by each of the methods [21]. Credibility of the modified Angoff method for the senior residents was judged by the number of PGY4&5 residents/orthopedic fellows passing the OSCE determined by each of the methods [20].

## Results

### Modified Angoff method by examiner type

A total of six sports surgeons, six non-sports surgeons, and six fellows undertook the modified Angoff method for the OSCE. A paired samples *t*-test showed that all examiner groups (based upon the mean for all three examiner types) expected senior residents to get a significantly higher percentage of checklist items correct compared to junior residents (all stations *p* < 0.001) (Table 1). While there was a tendency for non-sports surgeons to have the highest expectations for senior (and in some cases, junior) residents, there were no significant differences due to examiner type (all stations *p* >0.05). There was a high correlation (ICC) between judges for the modified Angoff method for both the junior residents (0.85) and for the senior residents (0.9).

**Table 1:** Modified Angoff method

Station	Residents	Sports Surgeon (95 % CI)	Non Sports Surgeon (95%CI)	Fellow (95 % CI)	Significance
1	Senior	0.57 (0.4-0.74)	0.64 (0.48-0.8)	0.58 (0.49-0.67)	*P* < 0.001
	Junior	0.37 (0.23-0.51)	0.37 (0.28-0.47)	0.33 (0.26-0.39)	
2	Senior	0.56 (0.37-0.75)	0.67 (0.44-0.9)	0.58 (0.47-0.69)	*P* < 0.001
	Junior	0.43 (0.26-0.6)	0.39 (0.25-0.52)	0.36 (0.26-0.46)	
3	Senior	0.46 (0.31-0.61)	0.57 (0.36-0.78)	0.49 (0.35-0.64)	*P* < 0.001
	Junior	0.35 (0.21-0.49)	0.34 (0.21-0.47)	0.29 (0.22-0.37)	
4	Senior	0.58 (0.27-0.76)	0.66 (0.42-0.89)	0.5 (0.37-0.63)	*P* < 0.001
	Junior	0.33 (0.18-0.49)	0.34 (0.2-0.49)	0.29 (0.24-0.35)	
5	Senior	0.56 (0.41-0.71)	0.71 (0.51-0.91)	0.56 (0.41-0.72)	*P* < 0.001
	Junior	0.38 (0.21-0.54)	0.41 (0.21-0.61)	0.36 (0.23-0.5)	
6	Senior	0.50 (0.35-0.65)	0.67 (0.49-0.84)	0.5 (0.31-0.69)	*P* < 0.001
	Junior	0.35 (0.21-0.5)	0.42 (0.27-0.57)	0.36 (0.24-0.48)	
Total	Mean Senior	0.53 (0.42-0.63)	0.65 (0.5-0.8)	0.54 (0.42-0.65)	*P* < 0.001
	Mean Junior	0.37 (0.27-0.47)	0.38 (0.27-0.49)	0.33 (0.26-0.41)	

Displayed are the expected checklist percentage correct by examiner type and by resident group (junior – PGY1-3, senior PGY 4&5). For all three groups of judges (six in each group), a significant difference was seen both overall and for each station between junior and senior residents (*p* < 0.001). No significant difference was seen between each group of judges. PGY – postgraduate year

### Differences between standard setting methods

The pass marks established by the BG method, BLR method and modified Angoff method are seen in Table 2 and 3. For senior residents, there were no significant differences between the pass marks determined by the modified Angoff method and the BG/BLR methods. For junior residents, there were significant differences for all stations and for the overall cut score, with the pass marks determined by the Angoff method significantly lower than the pass marks determined by the BG/BLR Method (all *p* < 0.001).

**Table 2:** Pass marks using modified Angoff for the senior residents, Borderline Groups (BG) and Borderline Regression (BLR) method

Station	Modified Angoff method (95 % CI)	Borderline groups method (95 % CI)	Borderline regression method (95 % CI)	Significance
1	0.6 (0.54-0.66)	0.53 (0.52-0.54)	0.52 (0.46-0.58)	n.s.
2	0.6 (0.52-0.68)	0.54 (0.53-0.56)	0.56 (0.51-0.61)	n.s.
3	0.51 (0.43-0.59)	0.48 (0.47-0.48)	0.47 (0.44-0.51)	n.s.
4	0.55 (0.46-0.65)	0.58 (0.49-0.66)	0.55 (0.46-0.64)	n.s.
5	0.61 (0.52-0.7)	0.54 (0.53-0.54)	0.56 (0.51-0.6)	n.s.
6	0.55 (0.46-0.64)	0.5 (0.48-0.51)	0.51 (0.47-0.54)	n.s.
Total	0.57 (0.49)-(0.65)	0.53 (0.5-0.55)	0.53 (0.43-0.62)	n.s.
Total + 1SEM	0.60	0.56	0.56	n.s.

No significant difference was seen on any station between the modified Angoff method and the BG/BLR methods for senior residents. *n.s*. non significant

**Table 3:** Pass marks using modified Angoff for the junior residents, Borderline Groups (BG) and Borderline Regression (BLR) method

Station	Modified Angoff method (95 % CI)	Borderline groups method (95 % CI)	Borderline regression method (95 % CI)	Significance
1	0.35 (0.31-0.4)	0.53 (0.52-0.54)	0.52 (0.46-0.58)	*P* < 0.001
2	0.39 (0.33-0.45)	0.54 (0.53-0.56)	0.56 (0.51-0.61)	*P* < 0.001
3	0.32 (0.27-0.37)	0.48 (0.47-0.48)	0.47 (0.44-0.51)	*P* < 0.001
4	0.32 (0.27-0.37)	0.58 (0.49-0.66)	0.55 (0.46-0.64)	*P* < 0.001
5	0.38 (0.31-(0.46)	0.54 (0.53-0.54)	0.56 (0.51-0.6)	*P* < 0.001
6	0.38 (0.31-0.44)	0.5 (0.48-0.51)	0.51 (0.47-0.54)	*P* < 0.001
Total	0.36 (0.3-0.42)	0.53 (0.5-0.55)	0.53 (0.43-0.62)	*P* < 0.001
Total + 1SEM	0.39	0.56	0.56	*P* < 0.001

A significant difference was seen for the modified Angoff and the BG/BLR methods for junior residents for all stations and overall (all *p* < 0.001)

**Fig. 1:**
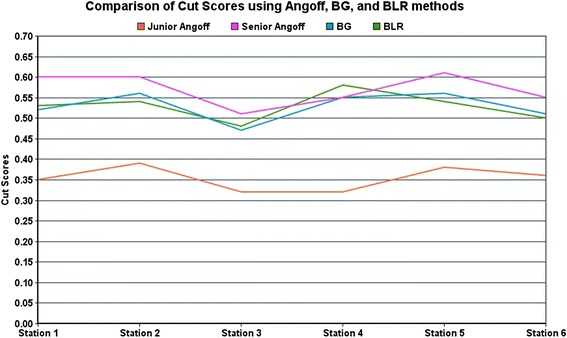
Comparison of cut-scores using the modified Angoff, Borderline Group (BG), and Borderline Regression (BLR) methods for junior and senior residents for each station

The number of residents who failed each station and failed overall using each standard setting method are seen in Table 4, with a comparison of each method seen in Fig. 1. Using the BG and the BLR method, 6/21 (28.6 %) of junior residents failed the OSCE; using the modified Angoff method, only 1/21 (4.8 %) junior residents failed. Using the BG, the BLR method, and the modified Angoff method, no senior resident or fellow failed the OSCE.

**Table 4:** Number of failures established by each standard setting method, for each station and overall

	Modified Angoff method			Borderline groups method			Borderline regression method		
Station	Junior (*n* = 21)	Senior (*n* = 18)	Fellow (*n* = 6)	Junior (*n* = 21)	Senior (*n* = 18)	Fellow (*n* = 6)	Junior (*n* = 21)	Senior (*n* = 18)	Fellow (*n* = 6)
1	1	1	1	4	0	0	4	0	0
2	0	2	1	6	1	1	6	1	1
3	0	2	1	11	2	0	11	2	0
4	1	0	0	9	0	0	9	0	0
5	2	1	0	8	0	0	7	0	0
6	4	2	4	11	1	4	10	0	3
**Overall**	**1**	**0**	**0**	**6**	**0**	**0**	**6**	**0**	**0**

Bold is the most important information

## Discussion

To our knowledge, this is the first study to examine the use of standard setting methods in the setting of postgraduate CBME. The results of this study demonstrate that the modified Angoff method can be used to establish different pass marks for junior and senior residents in the setting of CBME. We have also demonstrated that the subspecialty training of judges does not significantly change pass marks.

One of the challenges of the CBME model is the organization of frequent, objective assessments, requiring considerable faculty involvement and resources [22–24]. The curriculum at our institution has 21 different rotations – each resident is required to demonstrate a minimal level of competence in one rotation before progression to the next [25]. The sports medicine rotation is one of the major rotations, which residents undertake both as a junior and as a senior, with the curriculum map detailing different objectives for each group. While previous research had demonstrated that the junior residents could not master clinical skills as well as the seniors, we would prefer to continue using a single OSCE in the interest of feasibility (research will be published in December). Using the modified Angoff method to set different pass marks for junior and senior residents allows us to do so. In this manner, we can also identify the one or two junior residents who are performing poorly compared to their peers.

The advantages of the modified Angoff method include its relative simplicity, as well as the fact that the pass/fail standard can be set before the OSCE is undertaken [2]. Initially, the authors of this paper had been concerned that sports medicine specialists, who were involved in content review and exam writing for the sports rotation, would set the pass mark too high, especially for the junior residents. Interestingly, although there was no significant difference between the groups, there was a trend for the non-sports surgeons to demand more, especially from the senior residents. These results are encouraging; it seems logical to have those surgeons involved in the content development perform the standard setting procedure.

In 2000, Kaufman et al. compared the Angoff method (different from the modified Angoff, whereby judges estimate ‘the probability that a ‘minimally acceptable’ or borderline candidate would answer an item correctly’) [13] and the BG method, and found that both provide reasonable and defensible results in the medical school setting [6]. In contrast, Kramer et al. examined standard setting in postgraduate general practice training, identifying that the BLR method was more credible and acceptable than the modified Angoff method [20].

These conflicting findings may be the results of some known difficulties with the Angoff method. Verheggen et al. demonstrated considerable variation between judges, especially when judges had less expertise in certain item areas [18]. The authors recommended careful judge selection, and that judges should be capable of answering all items correctly. In the study by Kramer et al., 84 examiners were used, the majority of whom also performed the modified Angoff methods [20]. In our study only six judges were used, all of who were involved in exam creation and acted as OSCE examiners. This may explain why the modified Angoff method was shown to be credible and acceptable in our setting.

In this study, in order to perform the BG method and the BLR method, we were able to use the results of 45 participants, who sat the OSCE over 18 months, However, as an OSCE becomes a common assessment method within our CBME program, waiting to generate sufficient results before perform a BG/BLR method is not acceptable. For this reason, the findings that the pass marks established by the modified Angoff method are acceptable and credible, and can be performed by the subspecialty judges are extremely significant, and are of potential value to similar sized CBME based residency programs.

The results of the OSCE study demonstrated that while senior residents were able to achieve a minimal level of competence, junior residents were not (research to be published in December 2015). The results of this study are not dissimilar to studies that use progress testing, whereby regular assessment throughout an academic program are used to provide longitudinal evidence of the growth of student knowledge [26]. The sports medicine OSCE was not designed as a progress test, but rather to determine whether both senior and junior residents could achieve a minimal level of competence. However, continued iterations of the OSCE will be used in a manner similar to progress testing, to ensure that all residents are performing as expected in comparison with their peer group.

One of the main limitations of this study was an inability to demonstrate the credibility of using the modified Angoff to establish a pass mark for the junior residents. Credibility for the use of the modified Angoff to set pass marks for senior residents was established by a comparison with the pass marks established using the BLR/BG methods. However, in the setting of the junior residents, the pass mark created was significantly lower than that set by the BLR/BG methods. While some credibility for this standard setting method was demonstrated by the finding that all three groups of judges set similar cut scores for junior residents, there was no alternative standard setting method that could be used for comparison.

Downing wrote that there is no single correct answer when comparing standard-setting methods [21], with Norcini and Shea stating that issues of student fairness are the most important – the passing scores must be acceptable to students, faculty and administrators [16]. Having nearly 30 % of junior residents fail using the BG/BLR methods certainly appears unacceptable. The purpose of the end of rotation OSCE was to identify those residents not performing as well as their peer group, which the modified Angoff method appears to effectively do. Credibility can also be established by using a systematic approach, produced by qualified judges with a clear purpose [16], which was the case in the study. Methods should also be supported by a body of published research, be transparent, easy to implement and easy to explain – such methods justify the final result [15].

Other limitations of this study include the use of only six judges in each group to perform the modified Angoff method, despite evidence that increased number of judges improve the reliability of the modified Angoff – however there was a high correlation between judges for the modified Angoff, and the pass marks created for the senior residents matched that set by the BG/BLR method. In this study, the credibility as opposed to the validity of standard setting methods was studied, with credibility established by comparing the pass/fail rates of different methods with a reference group that is expected to have a high pass rate [20]. While these two terms could be used interchangeable, credibility is typically used in the standard setting literature and was thus used in this study [20]. Finally, this study also uses the OSCE results of relatively few residents, especially in comparison to other studies that have used the results of medical students - however it would be difficult to increase these numbers in the setting of postgraduate orthopedic training without performing a multicentred study.

## Conclusion

The results of this study demonstrate that the modified Angoff method can be used to set acceptable and credible cut-scores for junior and senior residents sitting an end of rotation OSCE in the setting of postgraduate CBME. This allows the use of the modified Angoff method to establish separate junior and senior cut-scores before each OSCE, not just in sports medicine, but also in other rotations such as joint replacement and trauma.
